# Balancing rice blast resistance and growth through suppression of the E3 ubiquitin ligase OsRING80

**DOI:** 10.1093/plphys/kiaf620

**Published:** 2025-11-26

**Authors:** Xinyi Hou, Xiaoman You, Xudong Mi, Xuanda Huang, Huiting Hu, Jisong Wang, Feng He, Ruyi Wang, Shutong Wang, Yuese Ning

**Affiliations:** College of Plant Protection, Hebei Agricultural University, Baoding 071000, China; State Key Laboratory for Biology of Plant Diseases and Insect Pests, Institute of Plant Protection, Chinese Academy of Agricultural Sciences, Beijing 100193, China; State Key Laboratory for Biology of Plant Diseases and Insect Pests, Institute of Plant Protection, Chinese Academy of Agricultural Sciences, Beijing 100193, China; State Key Laboratory for Biology of Plant Diseases and Insect Pests, Institute of Plant Protection, Chinese Academy of Agricultural Sciences, Beijing 100193, China; State Key Laboratory for Biology of Plant Diseases and Insect Pests, Institute of Plant Protection, Chinese Academy of Agricultural Sciences, Beijing 100193, China; State Key Laboratory for Biology of Plant Diseases and Insect Pests, Institute of Plant Protection, Chinese Academy of Agricultural Sciences, Beijing 100193, China; State Key Laboratory for Biology of Plant Diseases and Insect Pests, Institute of Plant Protection, Chinese Academy of Agricultural Sciences, Beijing 100193, China; State Key Laboratory for Biology of Plant Diseases and Insect Pests, Institute of Plant Protection, Chinese Academy of Agricultural Sciences, Beijing 100193, China; State Key Laboratory for Biology of Plant Diseases and Insect Pests, Institute of Plant Protection, Chinese Academy of Agricultural Sciences, Beijing 100193, China; College of Plant Protection, Hebei Agricultural University, Baoding 071000, China; State Key Laboratory for Biology of Plant Diseases and Insect Pests, Institute of Plant Protection, Chinese Academy of Agricultural Sciences, Beijing 100193, China

## Abstract

In crop plants, there is often a trade-off between growth and defense; modulating the balance is crucial for effective crop improvement. In rice (*Oryza sativa*), the *DENSE AND ERECT PANICLE 2* (*DEP2*) mutant shows altered phenotypes such as a more vertical leaf angle, erect panicles, small and round grains, slightly lower thousand-grain weight, and dwarfism. Here, we determined that loss of DEP2 function enhances resistance to the rice blast fungus *Magnaporthe oryzae*. We established that DEP2 interacts with the E3 ubiquitin ligase OsRING80, which ubiquitinates DEP2 and promotes its degradation via the 26S proteasome pathway. Interestingly, the transcriptional level of *OsRING80* was lower in *dep2* mutant lines, suggesting feedback regulation of OsRING80 by DEP2. Remarkably, knocking out *OsRING80* through CRISPR/Cas9-mediated gene editing resulted in greater resistance to *M. oryzae* without compromising plant growth. These findings reveal that OsRING80 plays a crucial role in balancing plant immunity and growth by targeting DEP2; they also provide a valuable candidate gene for developing improved rice cultivars with both disease resistance and optimal agronomic traits.

## Teaser

The E3 ubiquitin ligase OsRING80 plays a key role in maintaining the balance between plant immunity and growth by targeting DEP2, offering a promising strategy for breeding disease resistance crops.

## Introduction

Throughout the long-term co-evolution of plants and pathogenic micro-organisms, a 2-tiered defense system has developed to combat pathogen infection, including pattern-triggered immunity (PTI) and effector-triggered immunity (ETI) (Yuan et al. 2021a, 2021b). PTI refers to pathogen-associated molecular patterns (PAMPs), which are recognized by plants through pattern recognition receptors (PRRs) located on cell membranes. ETI is a defense mechanism by which plants detect pathogens directly or indirectly via intracellular NLR receptor proteins (Spoel and Dong 2012; Cui et al. 2015; Couto and Zipfel 2016; Yu et al. 2017). PRR usually senses pathogen or microbial-associated molecular patterns (PAMPs/MAMPs) and damage-associated molecular patterns (DAMPs) (Boller and Felix 2009; Boutrot and Zipfel 2017). These pathways result in numerous overlapping downstream effects, including mitogen-activated protein kinase (MAPK) cascades, calcium flux, reactive oxygen species (ROS) bursts, transcriptional reprogramming, and plant hormone signaling, indicating convergence points and intersections of these 2 signaling cascades (Tsuda and Katagiri 2010; Peng et al. 2018; Thulasi Devendrakumar et al. 2018; Yuan et al. 2021a, 2021b). But in plants, immunity to disease often leads to unexpected growth and yield losses (Ning et al. 2017). For example, in wheat, disruption of the susceptibility (*S*) gene, *Mildew Resistance Locus O* (*MLO*), confers plants broad-spectrum resistance to powdery mildew, but this resistance affects growth and causes yield loss (Büschges et al. 1997; Schulze-Lefert and Vogel 2000; Consonni et al. 2006). Achieving a balance between disease resistance and plant growth in plants is essential.

The ubiquitin proteasome system (UPS) is crucial for plant–microbe interactions and the immune response to pathogens (Zeng et al. 2006; Ning et al. 2016). As a highly specific posttranslational modification mechanism, it plays a crucial role in regulating gene function in rice. It is now known that ubiquitination is mediated by a cascade reaction of enzymes (E1, E2, and E3), that can deliver ubiquitin molecules in sequence to cellular targets (Pickart 2001; Pickart and Eddins 2004). The E3 ligase is a key factor in the selective degradation mechanism of the ubiquitin–proteasome system by recognizing the target protein and attaching ubiquitin to the substrate (Sadanandom et al. 2012). The UPS is a crucial pathway for the degradation of proteins in the cells and is recognized as a key regulator of plant immunity (Langin et al. 2020; Qu et al. 2021). By utilizing ubiquitination regulatory mechanisms to accurately modify target proteins, a balance might be achieved between disease resistance and growth mediated by these proteins.

Rice is a major global food crop, but rice blast, caused by *Magnaporthe oryzae*, is a serious threat to global rice production. During an epidemic of the disease, the yields are generally reduced by 10% to 30% (Devanna et al. 2022; Wang et al. 2025). Searching for resistance-related genes is crucial for disease resistance in rice. However, increasing resistance to rice blast through various defense mechanisms is often regarded as costly and there is considerable evidence that it can affect growth. For example, knockout of *SPL11* in rice results in reduced growth and increased resistance to rice blast (Fan et al. 2018). Similarly, inhibition of *APIP5* expression induces rice cell death and increases resistance to *M. oryzae* (Wang et al. 2016). Further study finds that the E3 ubiquitin ligase OsRING113 interacts with APIP5 and facilitates its degradation through the 26S proteasome pathway. Remarkably, overexpressing *OsRING113* provides broad-spectrum resistance to both *M. oryzae* and *Xanthomonas oryzae* pv. *oryzae* (*Xoo*) without inducing rice cell death (Zhang et al. 2024). Therefore, by identifying the associated E3 ligases, we might be able to fine-tune protein accumulation to achieve a balance between disease resistance and growth.

In rice, *DEP2* plays a critical role in the developmental process. *DEP2* can regulate panicle shape and seed size and is mainly expressed in young tissues such as young panicles. Compared to the wild type, the *dep2-2* mutant has a smaller leaf angle, an erect panicle, small and round grains, and a shorter plant (Abe et al. 2010; Li et al. 2010; Zhu et al. 2010; Zhu et al. 2021). In this study, we discovered that *dep2-2* mutant can enhance rice resistance to the blast pathogen *M. oryzae*, suggesting that *DEP2* acts as a negative regulator of the rice immune response. Our investigation revealed that the E3 ubiquitin ligase OsRING80 interacts with DEP2, facilitating its degradation via the 26S proteasome pathway. Notably, *osring80* enhances rice resistance to blast disease without impacting plant growth. Intriguingly, there appears to be a feedback regulation mechanism between DEP2 and OsRING80. In conclusion, the loss of *OsRING80* function improves rice resistance without incurring any growth penalties, highlighting OsRING80 as a promising candidate gene for developing resistant rice varieties.

## Results

### *DEP2* controls rice grain size and negatively regulates rice immunity against *M. oryzae*

**Figure 1.**
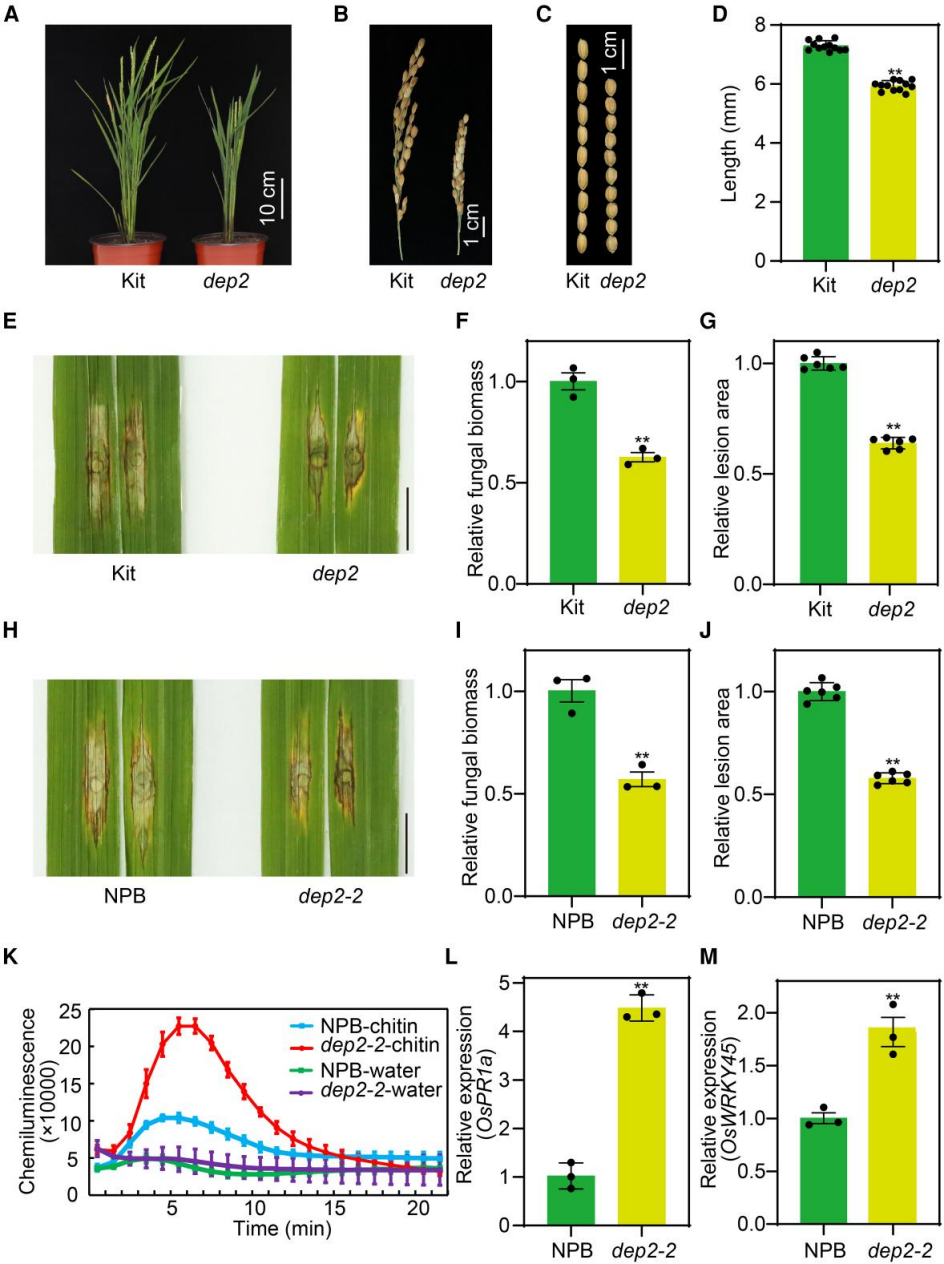
*DEP2* controls rice grain size and negatively regulates rice immunity against *M. oryzae*. **A** and **B)** Gross morphology **(A)** and panicle morphology **(B)** of Kit and *dep2* at the heading stage. Kit, Kitaake. **C** and **D**) Comparison of the grain length between the Kit plants and *dep2* mutant. **E** to **G)** Phenotypes of leaves from 6-wk-old *dep2* mutant inoculated with the compatible *M. oryzae* isolate RB22 **(E)**, relative fungal biomass, as determined by RT-qPCR **(F)**, and the relative lesion area, as measured using ImageJ **(G)**. Bars: 1 cm. **H** to **J)** Phenotypes of leaves from 6-wk-old *dep2-2* mutant inoculated with the compatible *M. oryzae* isolate RB22 **(H)**, relative fungal biomass, as determined by RT-qPCR **(I),** and the relative lesion area, as measured using ImageJ **(J)**. Bars: 1 cm. **K)** Chitin-induced ROS bursts in NPB and *dep2-2* leaves. ROS, reactive oxygen species. **L** and **M)** Relative transcript levels of the defense-related genes *OsPR1a*  **(L)** and *OsWRKY45*  **(M)** in *dep2-2* mutant. Data shown as means ± SD (*n* = 12, 3, 6, 3, 6, 3, 3, and 3 in **D**, **F**, **G**, **I**, **J**, **K**, **L**, and **M**, respectively). Asterisks represent statistically significant differences between the samples (**P < 0.01 by a Student's *t*-test).

A previous study identified *DEP2* as the gene responsible for erect panicle phenotype (Li et al. 2010). Interestingly, we found a phenotype similar to that of *dep2-2* mutant in the field (Li et al. 2010). We hypothesized that it might be caused by a *DEP2* mutation. Sequencing analysis revealed a single nucleotide deletion in the 7th exon of *DEP2*, leading to premature termination of the gene. Therefore, we confirmed the genotype of the *dep2* mutant (with 1-bp deletion of C) materials in the Kitaake (Kit) background (Supplementary Fig. S1). Similar to *dep2-2* mutant, *dep2* mutant also exhibited an erect panicle, a reduction in both plant height and panicle length (Fig. 1, A and B). And the mutant grains were shorter than the wild-type Kit (Fig. 1, C and D).

Studies have shown that there is often a trade-off between growth and defense in crops (He et al. 2022). So, we analyzed the expression patterns of *DEP2* gene in Nipponbare (NPB) rice plants infected with the compatible *M. oryzae* isolate RO1-1 and the incompatible *M. oryzae* isolate C9240. Reverse transcription quantitative PCR (RT-qPCR) analysis demonstrated that *DEP2* is slightly suppressed by *M. oryzae* infection (Supplementary Fig. S2). Based on this result, we speculated that *DEP2* might affect rice resistance. In order to evaluate the effect of *DEP2* on rice blast resistance, we used the punch inoculation method to assess the resistance of the *dep2* mutant under the Kit background to the compatible *M. oryzae* isolate RB22. Two weeks after inoculation with RB22, the *dep2* mutant exhibited increased resistance, characterized by smaller disease lesions and reduced fungal biomass (Fig. 1, E to G). To further validate this result, we used the same method to assess the resistance of the *dep2-2* mutant under the NPB background to the compatible *M. oryzae* isolate RB22 (Li et al. 2010). The survey showed that we got the same results (Fig. 1, H to J). On the contrary, the *proACTIN:SUG1* (OE-*DEP2*) plants (overexpressing *DEP2* in the ZH11 background) (Li et al. 2025) showed larger lesion area and were more susceptible to *M. oryzae* isolate RB22 (Supplementary Fig. S3, A to C). To investigate the role of DEP2 in PTI, we performed chemiluminescence analysis using chitin-treated 6-wk-old plants to detect ROS accumulation. ROS levels were higher in *dep2-2* mutant compared to NPB (Fig. 1K). In line with this result, RT-qPCR analysis revealed that the transcript levels of defense genes *OsPR1a* and *OsWRKY45* were significantly upregulated in *dep2-2* mutant than NPB (Fig. 1, L and M). Together, these results showed that DEP2 negatively regulates rice resistance to rice blast.

### DEP2 interacts with OsRING80

**Figure 2.**
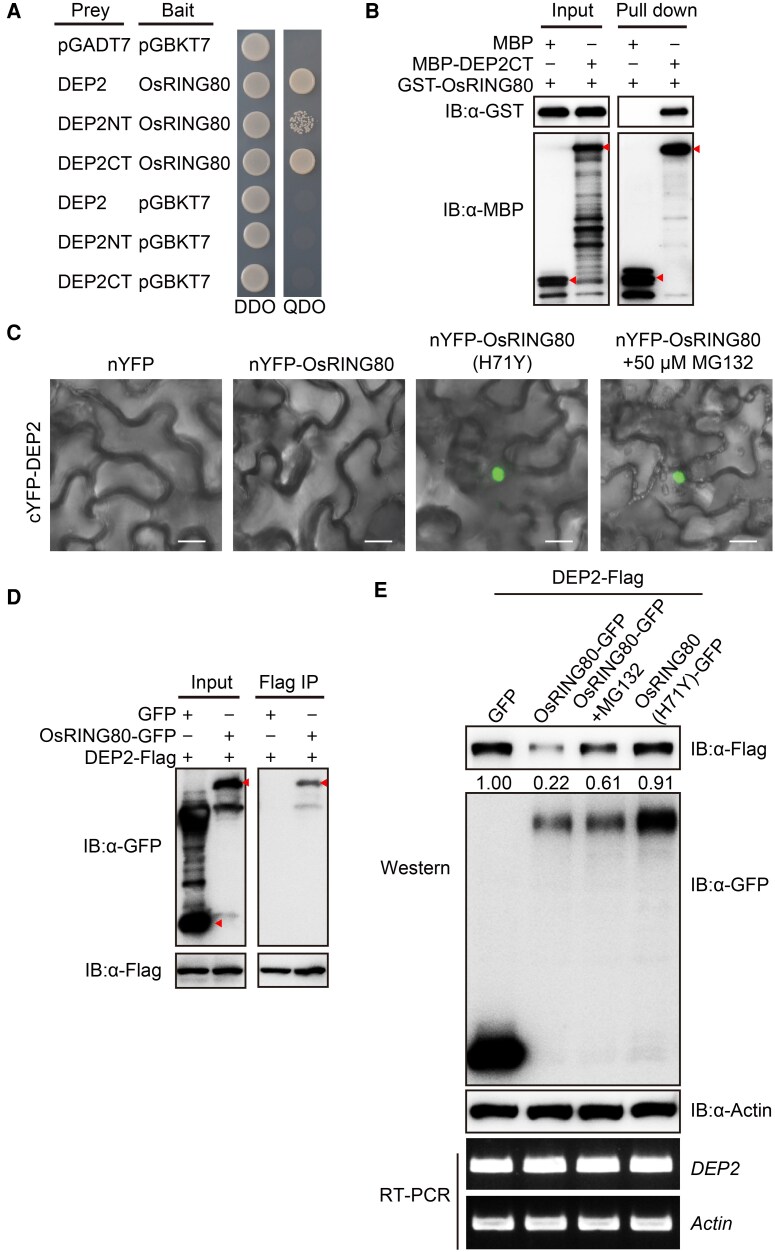
DEP2 interacts with and is ubiquitinated by OsRING80. **A)** Y2H assay showing the interaction between DEP2 and OsRING80. DDO (double dropout supplements: SD-Leu/-Trp), QDO (quadruple dropout supplements: SD-Ade/-His/-Leu/-Trp). NT, N terminal; CT, C terminal. **B)** Pull-down assay to detect the interaction of DEP2 and OsRING80. MBP was used as negative control. Red arrowheads indicate the expected proteins. MBP, maltose-binding protein; GST, glutathione S-transferase. **C)** BiFC assay to test the interaction between DEP2 and OsRING80 in *N. benthamiana* leaves. Bars: 20 μm. **D)** Co-IP assay to test the interaction between DEP2 and OsRING80 in *N. benthamiana* leaves. Total protein was extracted from *N. benthamiana* leaves transfected with the indicated constructs. Red arrowheads indicate the expected proteins. **E)** OsRING80 promotes the degradation of DEP2 via the 26S proteasome pathway in *N. benthamiana*. DEP2-Flag was co-expressed with OsRING80-GFP and OsRING80 (H71Y)-GFP in *N. benthamiana*. Tissues were harvested 2 days after infiltration. The 26S proteasome inhibitor MG132 (50 μM) was infiltrated into the tissues at 24 h before sampling, with DMSO used as a control.

To explore the homeostatic regulatory mechanisms of *DEP2* in rice resistance, we screened the UbE3-ORFeome library using the DEP2's C-terminus protein as a bait (Wang et al. 2022) (Supplementary Fig. S4), and identified potential interaction E3 ubiquitin ligase. Among the candidate interacting proteins, OsRING80, a SINA-type E3 ubiquitin ligase formerly called OsDIS1, caught our attention (Ning et al. 2011). To further verify the interaction between DEP2 and OsRING80, we co-transformed the yeasts with plasmids encoding these proteins and results showed that the full length DEP2, as well as its C-terminus or N-terminus can interact with OsRING80 (Fig. 2A). The pull-down assay showed that DEP2 directly binds to OsRING80 in vitro (Fig. 2B). We also performed bimolecular fluorescence complementation (BiFC) assays in *Nicotiana benthamiana* leaves. After infiltration, no fluorescence signals were observed in leaves co-expressing nYFP-OsRING80 and cYFP-DEP2. However, fluorescent signals were both detected following treatment with the 26S proteasome inhibitor MG132 and co-expression of cYFP-DEP2 and nYFP-OsRING80(H71Y) (Fig. 2C), a mutant that causes a loss of E3 ligase activity of OsRING80 (Ning et al. 2011). These results suggest that the interaction between DEP2 and OsRING80 occurs at the nucleus and that OsRING80 might promote the degradation of DEP2 *in planta*. Furthermore, we performed a co-immunoprecipitation (Co-IP) assay in *N. benthamiana*. OsRING80-GFP was specifically identified in the immune complex containing DEP2-Flag by western blot (Fig. 2D). Taken together, these results suggest that DEP2 interacts with OsRING80 in vitro and in vivo.

### DEP2 is degraded by OsRING80 via the ubiquitin 26S proteasome

Consistent with previous study, an in vitro ubiquitination assay demonstrated that OsRING80 possesses E3 ubiquitin ligase activity (Ning et al. 2011). To confirm that OsRING80 facilitates the degradation of DEP2 through ubiquitination, we co-expressed DEP2-Flag with either OsRING80-GFP or OsRING80(H71Y)-GFP in *N. benthamiana* leaves. The results showed that the intensity of DEP2-Flag bands remained similar when DEP2-Flag was co-expressed with GFP or OsRING80 (H71Y)-GFP. However, when DEP2-Flag was co-expressed with OsRING80-GFP, the intensity of DEP2-Flag bands was significantly reduced, and this suppression in DEP2-Flag accumulation was recovered with MG132 treatment (Fig. 2E). The internal control, ACTIN, displayed consistent accumulation across all tested combinations. *DEP2* and *ACTIN* were also expressed at similar levels in all combinations as well, as determined by RT-PCR (Fig. 2E). These results suggest that the E3 ligase OsRING80 specifically promotes DEP2 degradation via the 26S proteasome-dependent pathway *in planta*.

### The rice *osring80* mutant enhanced resistance against *M. oryzae*

**Figure 3.**
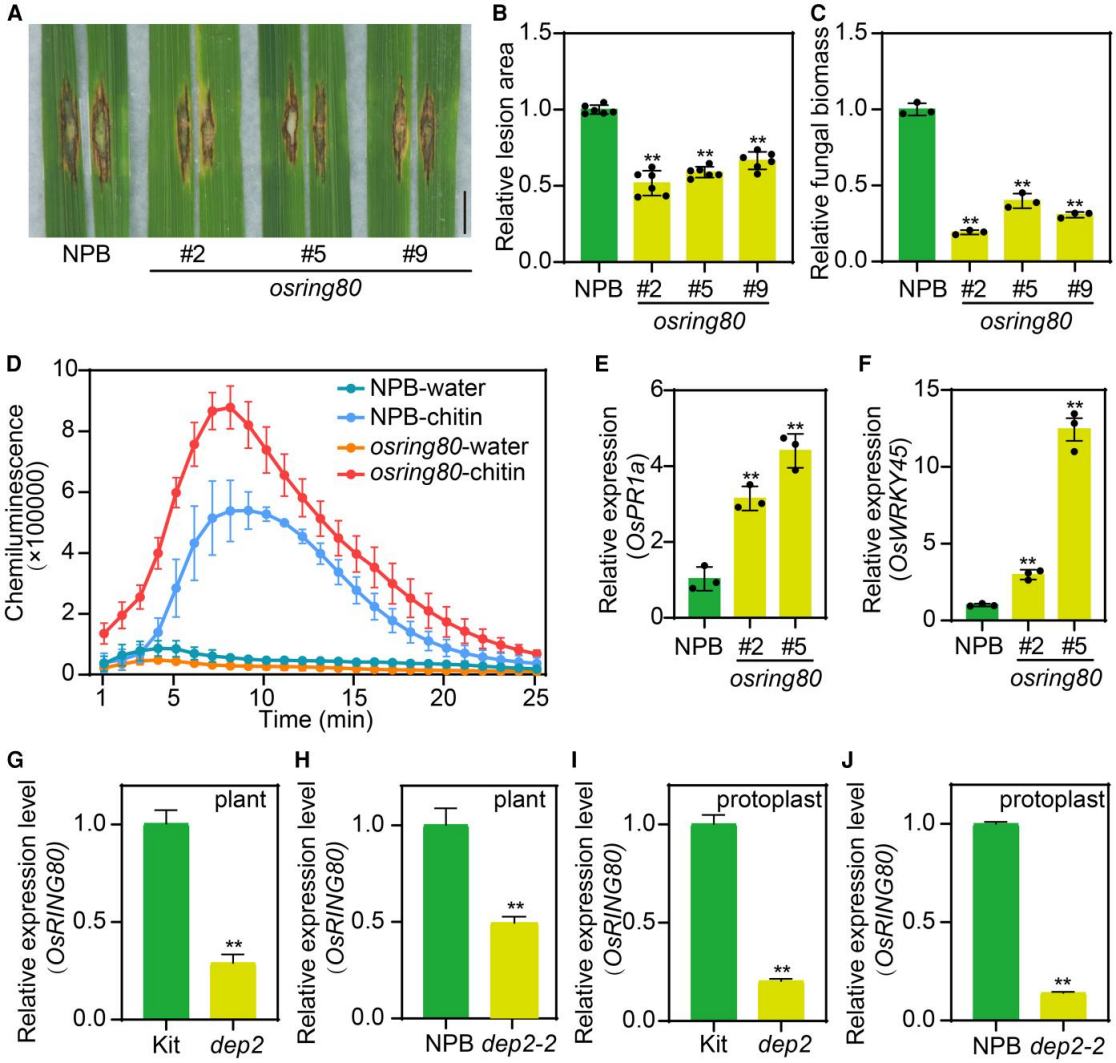
*OsRING80* negatively regulates rice immunity against *M. oryzae*. **A)** Phenotypes of the leaves from 6-wk-old *osring80* mutant inoculated with the compatible *M. oryzae* isolate RB22. Bars: 1 cm. **B)** Relative lesion area in NPB and *osring80* mutant after punch inoculation with *M. oryzae* isolate RB22. **C)** Relative fungal biomass in NPB and *osring80* mutant after punch inoculation with *M. oryzae* isolate RB22. **D)** Chitin-induced ROS bursts in NPB and *osring80* leaves. ROS, reactive oxygen species. **E** and **F)** Relative transcript levels of the defense-related genes *OsPR1a*  **(E)** and *OsWRKY45*  **(F)** in *osring80* mutant plants. **G)** The relative expression level of *OsRING80* in the Kit and *dep2* lines as determined by RT-qPCR. **H)** The relative expression level of *OsRING80* in the NPB and *dep2-2* lines. **I)** The relative expression level of *OsRING80* in the Kit and *dep2* rice protoplast. **J)** The relative expression level of *OsRING80* in the NPB and *dep2-2* rice protoplast. Data shown as means ± SD (*n* = 6, 3, 3, 3, 3, 3, 3, 3, and 3 in **B**, **C**, **D**, **E**, **F**, **G**, **H**, **I**, and **J**, respectively). Asterisks represent statistically significant differences between the samples (**P < 0.01 by a Student's *t*-test).

To determine whether *OsRING80* is involved in disease resistance in rice, we examined its expression pattern in NPB plants infected with RO1-1 and C9240. Result showed that *OsRING80* is slightly induced following inoculation compared to mock-inoculated plants (Supplementary Fig. S5). To explore the role of *OsRING80* in rice resistance, we generated *OsRING80* knockout mutant using CRISPR/Cas9-mediated gene editing. After genotyping, we selected 3 independent homozygous mutant lines (#2, #5, and #9; with 1-bp insertion of T, 8-bp deletion, and 1-bp insertion of A, respectively, all resulting in frameshift mutations) were selected for inoculation assays (Supplementary Fig. S6). Two weeks after inoculation with RB22, the *osring80* mutant exhibited smaller disease lesions and accumulated less fungal biomass than NPB (Fig. 3, A to C). Meanwhile, we generated *OsRING80* overexpression lines (OE-*OsRING80*, lines #11, #13 and #17) (Supplementary Fig. S7A). OE-*OsRING80* plants showed larger lesion area and were more susceptible to *M. oryzae* isolate RB22 (Supplementary Fig. S7, B to D). To investigate the role of OsRING80 in PTI, we performed chemiluminescence analysis using chitin-treated 6-wk-old plants to detect ROS accumulation. ROS levels were higher in *osring80* mutant compared to NPB (Fig. 3D). Consistent with the disease-resistant phenotype observed in the mutant, the expression levels of the defense-related genes *OsPR1a* and *OsWRKY45* were significantly higher in *osring80* mutant compared to NPB plants (Fig. 3, E and F). Interestingly, OsRING80 can degrade DEP2 via the 26S proteasome pathway. However, the mutant corresponding to these 2 genes exhibited consistent phenotypic characteristics. In view of this, we conducted an in-depth analysis of the transcriptional level of *OsRING80* in the *dep2* mutant by referring to the research approach of the SPIN6-SPL11 (Liu et al. 2015). Consequently, it was found that the level of *OsRING80* expression was reduced in both *dep2* mutant and *dep2-2* mutant (Fig. 3, G and H). We then expressed *OsRING80* in the rice protoplasts isolated from *dep2* mutant and NPB plants and found that *OsRING80* expression was also reduced (Fig. 3, I and J). Based on this, we speculated that there might be a feedback regulatory mechanism between DEP2 and OsRING80. Taken together, these findings manifest that *OsRING80* negatively regulates the disease resistance in rice and is potentially under the feedback regulatory control of *DEP2*.

### The enhanced disease resistance to rice blast in *osring80* mutant does not affect their major agronomic traits

**Figure 4.**
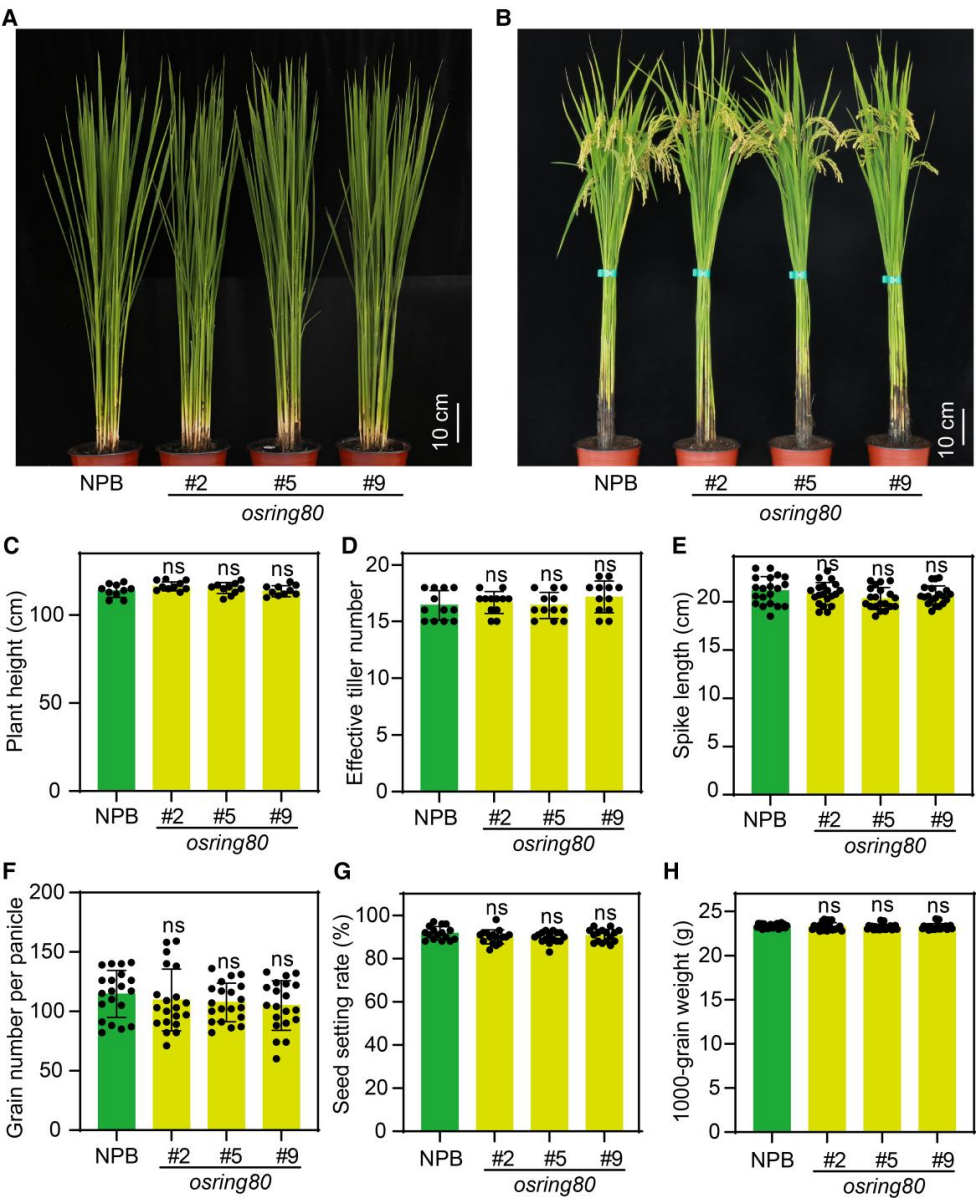
Major agronomic traits measured in *osring80* mutant lines. **A)** Gross morphology of *osring80* mutant and NPB plants at the tillering stage. **B)** Gross morphology of *osring80* mutant and NPB plants at the heading stage. **C** to **H**) Major agronomic traits measured: plant height **(C)**, effective tiller number **(D)**, spike length **(E)**, grain number per panicle **(F)**, seed setting rate **(G)**, and 1,000-grain weight **(H)**. Data shown as means ± SD (*n* = 10, 12, 20, 20, 16, and 23 in **C** to **H**, respectively). “ns” indicates no statistical significance at *P* > 0.05 according to Student's *t*-test.

To assess whether the enhanced disease resistance of the *osring80* mutant affects rice growth, we evaluated the main agronomic traits of the wild type and the mutant. NPB plants and *osring80* mutant were similar in terms of plant architecture and spike shape type (Fig. 4, A and B). The *osring80* mutant grains showed no significant difference to the NPB (Supplementary Fig. S8, A and B). There were also no significant differences in yield-related traits (plant height, effective tiller number, spike length, grain number per panicle, seed setting rate, and 1,000-grain weight) among the genotypes (Fig. 4, C to H). These results suggest that the loss of OsRING80 function does not cause a growth penalty in rice.

## Discussion

### DEP2 negatively regulates rice immune responses that affect rice yield

The process of plant disease resistance activates various defense responses that consuming significant amounts of energy and metabolites, which often affects the distribution of these resources, leading to a trade-off between plant growth and development and disease resistance (He et al. 2022). For example, knockout of *ROD1* gene in rice can enhance resistance to multiple pathogens; however, it also results in a reduces yield (Gao et al. 2021). Similarly, the *rbl1* mutant exhibits broad-spectrum disease resistance, but their yield is extremely low (Sha et al. 2023). Plant type is an important agronomic trait and a key factor in determining the high yield of rice. The erect spike in rice is one of the most important agronomic traits influencing grain yield. Previous research has identified 2 rice mutant with erect spikes, named *dep2-1* and *dep2-2* (Li et al. 2010). DEP2 plays a crucial role in determining the shape of rice spikes and seed size. It is predominantly expressed in young tissues, including developing spikes (Li et al. 2010; Yu et al. 2017). Compared to the wild type, *dep2* spikes were more erect and the grains were smaller, resulting in a slight reduction in 1,000-grain weight. Consequently, upon observing a change in the yield of the *dep2* mutant, this study thoroughly investigated the role of *DEP2* in rice immunity and its regulatory mechanisms. Then we found that knocking out the *DEP2* gene enhanced rice resistance to rice blast (Fig. 1, E to J). In addition, chitin-induced ROS accumulation and the upregulated expression of defense-related genes suggest that *DEP2* negatively regulates plant immunity through the PTI pathway. Typically, there is a trade-off exists between plant development and disease resistance (He et al. 2022). These results suggest that DEP2 is unable to balance disease resistance and growth. It is essential for plant growth and development and might also negatively regulate the basal defense of rice against rice blast.

### DEP2 interacts with and is ubiquitinated by OsRING80

Plant structure is a complex trait that has a significant impact on crop performance and productivity. Currently, erect panicle varieties are extensively cultivated in *japonica* rice-growing areas in China. In rice, *DEP2* plays a crucial role in regulating panicle type and seed size. DEP2, as a transcriptional regulator, can affect the developmental process of leaf tilting by regulating the transcriptional activity of *OsLIC* (Zhu et al. 2021), but its homeostatic regulatory mechanism is not yet clear. In this study, we screened an E3 ubiquitin ligase OsRING80 using DEP2CT as a bait. We then confirmed that OsRING80 can interact with DEP2 in vitro and in vivo (Fig. 2, A to D). OsRING80, an E3 ubiquitin ligase of the rice SINA family, has been shown to regulate the drought stress response of rice through protein interactions with OsNek6 (Ning et al. 2011). In this study, we found that OsRING80 facilitates the degradation of DEP2 through the 26S proteasome pathway and *osring80* mutant exhibits enhanced resistance to rice blast (Figs. 2E and 3). These results suggest that OsRING80 might play a role in abiotic and biotic stress by targeting different substrates.

### OsRING80 balance rice disease resistance and growth

Recent studies have shown that regulating certain genes can effectively balance plant growth and defense mechanisms (Gao et al. 2024). For example, OsUBC45, a ubiquitin-conjugating enzyme, can enhance rice yield and broad-spectrum disease resistance simultaneously for sustainable rice production (Wang et al. 2023). In this study, we found that DEP2 is unable to balance disease resistance and growth. However, we identified an E3 ubiquitin ligase, OsRING80, which can regulate DEP2. Based on this finding, we proceeded to generate *osring80* mutant. Through inoculation assays, we found that the loss of *OsRING80* function enhanced resistance to *M. oryzae* (Fig. 3, A to C). Furthermore, the expression levels of defense-related genes were elevated in *osring80* mutant. Results showed that *OsRING80* is a negative regulator of rice blast disease as well as *DEP2*. Previous study has found that the E3 ligase SPL11 can degrade SPIN6, but both negatively regulate rice immunity, and further studies revealed that the transcript level of *SPL11* was significantly suppressed in *SPIN6* RNAi plants, suggesting a feedback regulation between *SPIN6* and *SPL11* (Liu et al. 2015). Similarly, our findings revealed that *OsRING80* transcription was significantly down-regulated in *dep2* mutant and *dep2-2* mutant, indicating that DEP2 exerts feedback regulation on OsRING80. Consequently, we suspect that there are additional, yet-to-be-clarified molecular mechanisms linking the 2 genes. These results indicate that DEP2 negatively regulates rice blast and disease defense by directly related to OsRING80.

Notably, in the *osring80* mutant, several key agronomic traits, including plant height and 1,000-grain weight, remained similar to those of the wild type (Fig. 4, A to H and Supplementary Figs. S8, A and B). Interestingly, the experimental results showed that, unlike the erect panicle phenotype of the *dep2* mutant, the panicle shape of the *osring80* mutant was not significantly changed. This difference suggests that OsRING80 is only involved in the DEP2-mediated disease resistance signaling pathway, indicating that the growth and development of DEP2 may be regulated by other proteins. Our findings suggest that OsRING80, an E3 ubiquitin ligase, is able to regulate DEP2 through the ubiquitination modification pathway to striking a balance between disease resistance and growth. Simultaneously, the feedback regulation between DEP2 and OsRING80 might also enhance immunity in the *osring80* mutant.

**Figure 5.**
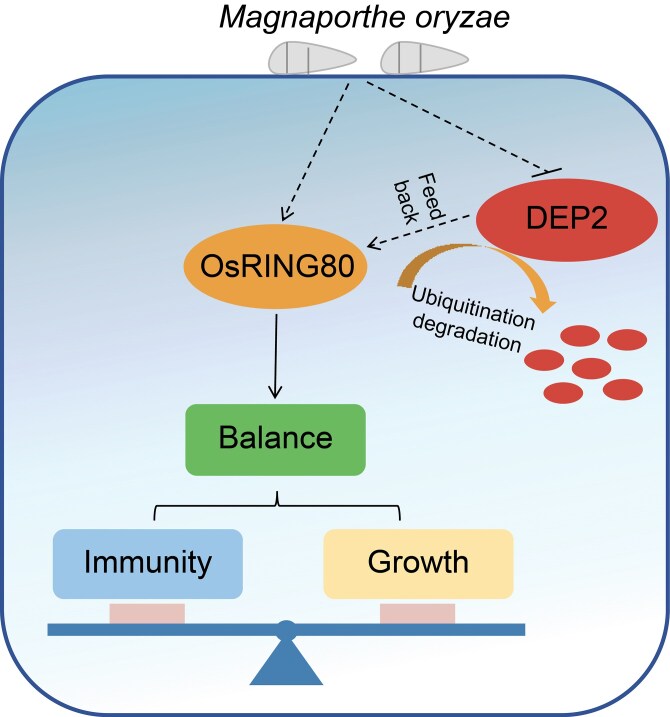
A proposed working model for the role of OsRING80 in balancing plant defense and growth. *OsRING80* expression is induced by *M. oryzae*, while *DEP2* expression is slightly suppressed. DEP2 is ubiquitinated and degraded by the E3 ligase OsRING80 through the 26S proteasome system. At the same time, *DEP2* maybe feedback regulates *OsRING80*. *OsRING80* can balance plant growth and defense response.

Based on the above results, we proposed a working model to demonstrate how OsRING80 and DEP2 interact to regulate immunity in rice (Fig. 5). DEP2 is ubiquitinated and subsequently degraded by the E3 ligase OsRING80 through the 26S proteasome system. Simultaneously, DEP2 might be able to feedback control OsRING80. The expression level of the *PR* genes was significantly upregulated in the *osring80* mutant. Notably, the *osring80* mutant improved resistance to rice blast without significantly affecting normal rice growth and development. These results demonstrate that *OsRING80* is an extremely promising candidate for genome editing. The discovery of the role of *OsRING80* provides an innovative strategy for developing disease-resistant rice varieties, paving the way for improved resistance to pathogens.

## Materials and methods

### Plant materials

The rice (*Oryza sativa*) cultivars NPB and Kit were used for all experiments in this study. And the transgenic rice in the NPB background was generated using *Agrobacterium*-mediated transformation of rice calli as previously described (Wang et al. 2016). Rice seeds were surface-sterilized by immersing in 75% (vol/vol) ethanol for 5 min, followed by 25 min immersion in 40% (vol/vol) sodium hypochlorite. The seeds were rinsed 6 to 8 times with sterile water and then germinated on 1/2 Murashige Skoog medium for 1 wk. After spending a week in the incubator, the seedlings were moved to soil in a growth chamber.

*N. benthamian*a plants were cultivated in soil under 12-h photoperiod at 25 °C. After 5 wk, *N. benthamiana* leaves can be used for Co-IP and BiFC assays.

### Rice blast inoculation and disease symptom evaluation

Rice leaves were inoculated by punching, as previously described with slight modifications (Wang et al. 2021). Six-week-old rice leaves were used for punch inoculation with *M. oryzae* spores (about 5 × 10^5^ conidia/mL), which were cultivated under weak light for 2 wk to spores induction. Then a mouse ear clip was used to lightly punch rice leaves, 10 μL of spore suspension was treated onto each punched site. The spores were held in place by sealing both sides of the treated sites with scotch tape. Disease symptoms on leaves were surveyed after inoculation 2 wk. DNA-based quantitative PCR (qPCR) was used to determine the relative fungal biomass by using the threshold period value (CT) of *M. oryzae MoPot2* gene and the CT value of rice ubiquitin gene (Shi et al. 2018). All inoculation experiments were independently repeated 3 times. Primers used are listed in Supplementary Table S1.

### RNA extraction and RT-qPCR

Total RNA was extracted from the sample using the UNIQ-10 column TRIzol Total RNA separation kit (Biotech). Using a transgenic 1-step gDNA removal and cDNA synthesis super hybrid kit (transgenic biotechnology). With 1 μL of 1:5 diluted cDNA as the template. RT-qPCR was performed using the SYBR qPCR Master Mixing Kit (Vazyme) and ABI Prism 7500 real-time fluorescent quantitative PCR system. *ACTIN* (entry number AK100267) was used as the internal reference gene. Primers used are listed in Supplementary Table S1.

### Yeast 2-hybrid assays

The full-length coding sequences or specific domains of *DEP2* and *OsRING80* were amplified and cloned into pGADT7 or pGBKT7 (Clontech) vectors. Follow the manufacturer's instructions (Cloning Technology Yeast Protocol Manual) to co-transform the constructs to the yeast strain AH109 (Wang et al. 2022). Primers used to generate the yeast 2-hybrid (Y2H) structure are listed in Supplementary Table S1.

### ROS detection

The ROS detection method was described previously (He et al. 2025). Disks were punched on the leaves of 6-wk-old plants and incubated in sterile water in the dark for 12 h. Three leave disks were immersed in a 1.5-mL centrifuge tube containing 1 μg of horseradish peroxidase (Jackson ImmunoResearch), 100 μL of luminol (Bio-Rad Immun-Star horseradish peroxidase substrate 1705040), and 1 μL of 8 μM chitin (hexa-*N*-acetyl-chitohexaose), and 1 μL of distilled water (which used as the control). Immediately after mixing the above ingredients, the test tube was placed in the GloMax 20/20 photometer (Promega) and the luminescence was measured for 22 min at 1-min intervals. The gene expression was obtained by repeated technique 3 times.

### Expression pattern analysis

After spraying rice leaves with different blast fungi, rice leaves were collected at different time points. Water containing 0.05% (vol/vol) Tween 20 was used as a Mock inoculation control (Fang et al. 2021). Then the RNA was extracted to synthesize cDNA. RT-qPCR was performed using SYBR qPCR Master Mixing Kit (Vazyme) and ABI Prism 7500 real-time fluorescent quantitative PCR system. The gene expression was obtained by repeated technique 3 times. Primers used are listed in Supplementary Table S1.

### In vitro pull-down assay

To generate GST and MBP labeled fusion proteins, we amplified the coding sequences for *OsRING80* and *DEP2* and inserted them into pGEX4T-2 or pMAL-c2X. The pull-down test was performed as previously described (Cai et al. 2021). After cell lysis, GST-OsRING80 was mixed with an equal amount of the MBP or MBP-DEP2CT. The mixture is then added to 1 mL of PBS buffer. The mixture was shaken gently at 4 °C for 1 h, then 20 to 30 μL amylose resin (E8021S, NEB) was added, and incubated at 4 C for 1 h. Then wash the beads 3 times with PBS. The fusion proteins of anti-GST (AbM59001-2H5-PU, BGI Genomics) and anti-MBP (A02070-2, Abbkine) antibodies were detected by western blot. Primers used are listed in Supplementary Table S1.

### In vivo Co-IP assay

The full-length coding sequences of *DEP2* and *OsRING80* were amplified and cloned into the pCAMBIA1300-221-Flag or pCAMBIA1305-GFP vector. The plasmids were transiently expressed in *N. benthamiana* leaves via *Agrobacterium*-mediated transfection. Two days after transfection, total proteins were extracted from freshly harvested leaves in 2 volumes of NB1 buffer (50 mM Tris-MES at pH 8.0, 0.5 m sucrose, 1 mM MgCl_2_, 10 mM EDTA, 5 mM DTT, and protease inhibitor cocktail) and incubated at 4 °C for 30 min with rotation. Co-IP assays were performed as previously described (Wang et al. 2016). The protein was gently incubated with an anti-Flag antibody at 4 °C for 1 h, then pre-washed protein G beads (micropores) were added to the protein antibody samples, and incubated for another 2 h. Then wash with protein extraction buffer 3 times and boil with 100 μL 1× SDS loading buffer at 100 °C for 5 min. The proteins were then detected with anti-Flag and anti-GFP. Antibodies used are listed in Supplementary Table S2.

### BiFC assay

For the BiFC assay, the full-length coding sequences of *OsRING80* and *DEP2* were fused to the p2YC (cYFP) or p2YN (nYFP) vector. The recombinant plasmids were transformed into *Agrobacterium* EHA105 and then co-permeated as previously described (You et al. 2019). *Agrobacterium* cultures individually containing the respective constructs were adjusted to an OD_600_ of 0.6 to 1.0 with MES buffer (10 mM MgCl_2_, 10 mM MES, and 0.2 mM acetosyringone, pH 5.6). The *N. benthamiana* leaves were co-permeated with the mixture. After 48 to 72 h of injection, the fluorescence signal was detected by laser scanning confocal microscopy (Zeiss LSM880).

### In vivo degradation assay

The in vivo OsRING80-DEP2 degradation assay was performed as previously described (You et al. 2023). The DEP2-Flag plasmid was co-transfected with OsRING80-GFP and OsRING80(H71Y)-GFP plasmids into *N. benthamiana* leaves via *Agrobacterium*-mediated transfection. Samples were collected for protein and RNA extraction at 48 h after infiltration. 50 μM MG132 was injected into the leaves 24 h before sampling; DMSO was used as a control. The protein and RNA of the sample were extracted 48 to 72 h after transfection. MG132 was injected 24 h before sample collection and DMSO was used as blank control.

### Quantification and statistical analysis

Statistical parameters are reported in the figures and figure legends. RT-qPCR, fungal biomass, and ROS assays were measured by 3 repetitions, and bars represent means ± SE. The asterisks indicate significant differences from the controls by 2-tailed Student's *t*-test (*P < 0.05, **P < 0.01).

### Accession numbers

The entry number sequence data mentioned in this paper can be found in the Rice Genome Annotation Project under the following accessions: *DEP2*, *LOC_Os07g42410*; *OsRING80*, *LOC_Os03g24040*.

## Supplementary Material

kiaf620_Supplementary_Data

## Data Availability

All data are incorporated into the article and its online supplementary material.

## Supplementary data

The following materials are available in the online version of this article.

**Supplementary Figure S1.** Schematic representation of the *DEP2* gene structure.

**Supplementary Figure S2.** Expression profile of *DEP2* during *M. oryzae* infection.

**Supplementary Figure S3.**  *DEP2* negatively regulates rice immunity against *M. oryzae*.

**Supplementary Figure S4.** Diagram of the domain structure of DEP2.

**Supplementary Figure S5.** Expression profile of *OsRING80* during *M. oryzae* infection.

**Supplementary Figure S6.** CRISPR/Cas9 mediated mutation of *osring80*.

**Supplementary Figure S7.**  *OsRING80* negatively regulates rice immunity against *M. oryzae*.

**Supplementary Figure S8.** The grain length phenotype of the *Osring80* mutant.

**Supplementary Table S1.** Sequences of the primers used in this study.

**Supplementary Table S2.** Antibodies used in this study.

## Dive Curated Terms

The following phenotypic, genotypic, and functional terms are of significance to the work described in this paper:

MES CHEBI: CHEBI:39010

GFP Gramene: green fluorescent protein
GFP Araport: green fluorescent protein
