# Supplementary material for: Balancing rice blast resistance and growth through suppression of the E3 ubiquitin ligase OsRING80
Source: Plant Physiol. 2025 Nov 26;199(4):kiaf620. doi: 10.1093/plphys/kiaf620 (PMC12715182; doi:10.1093/plphys/kiaf620)
Supplement: kiaf620_Supplementary_Data [file kiaf620_supplementary_data.pdf]

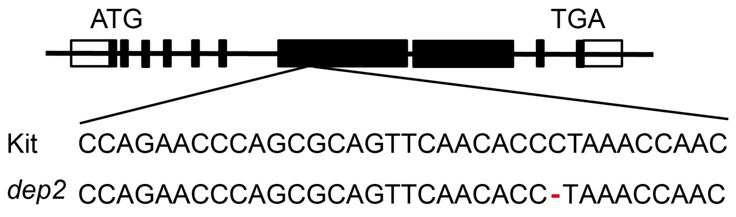

Supplementary Figure S1. Schematic representation of the *DEP2* gene structure.
Black boxes indicate the coding sequence, white boxes indicate the 5′ and 3′ untranslated regions, and lines between boxes indicate introns.

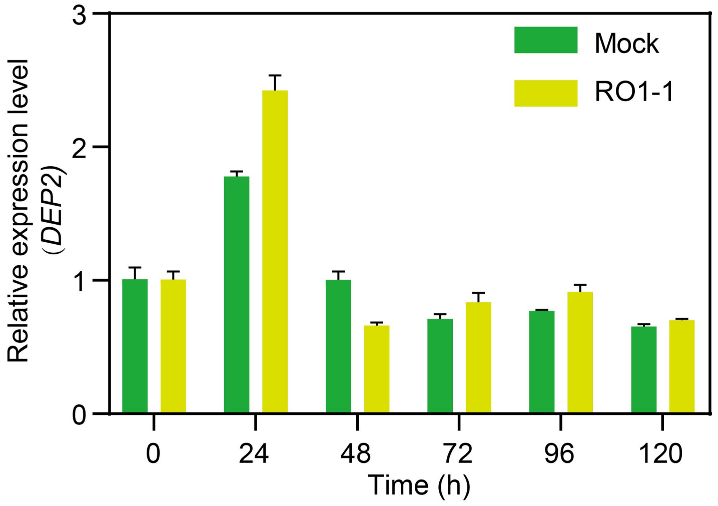

Supplementary Figure S2. Expression profile of *DEP2* during *M. oryzae* infection. *DEP2* expression in Nipponbare rice plants inoculated with the compatible *M. oryzae* isolate RO1-1 or the incompatible *M. oryzae* isolate C9240, as determined by RT-qPCR. ddH_2O was used as a mock-inoculation control, and rice *UBIQUITIN* (*UBQ*) was used as a reference gene to normalize gene expression. Data shown as means ± SD (n = 3).

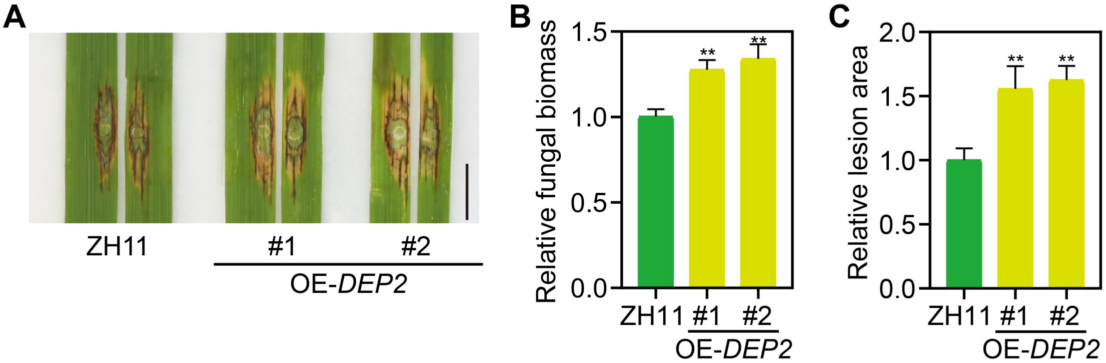

Supplementary Figure S3. *DEP2* negatively regulates rice immunity against *M. oryzae.*
A-C) Phenotypes of leaves from 6-week-old OE-*DEP2* materials inoculated with the compatible *M. oryzae* isolate RB22 (A), relative fungal biomass, as determined by RT-qPCR (B), and the relative lesion area, as measured using ImageJ (C). Data shown as means ± SD (n = 3). Asterisks represent statistically significant differences between the samples (**P < 0.01 by a Student's t-test). Bars: 1 cm.

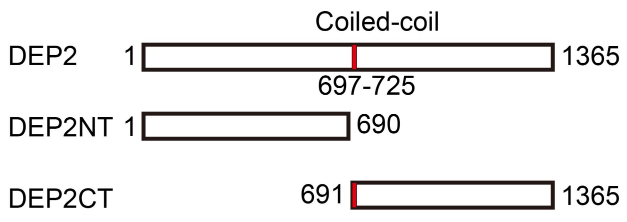

Supplementary Figure S4. Diagram of the domain structure of DEP2.
DEP2 full length (1–1365 aa), DEP2 N-terminal (1–690 aa), DEP2 C-terminal (691–1365 aa).

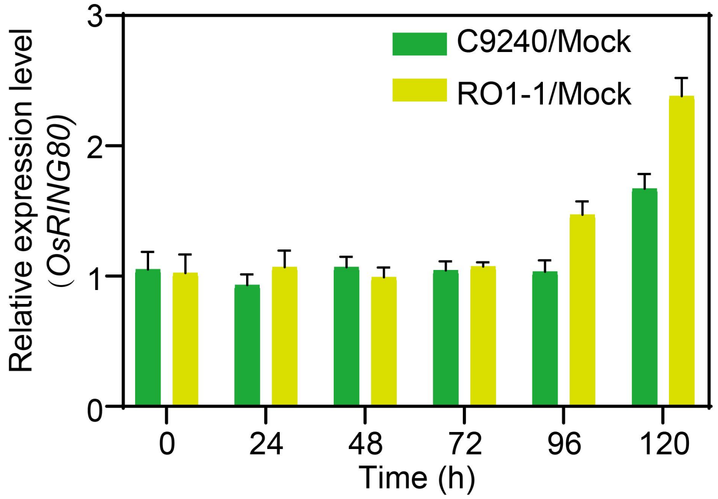

Supplementary Figure S5. Expression profile of *OsRING80* during *M. oryzae* infection. *OsRING80* expression in Nipponbare rice plants inoculated with the compatible *M. oryzae* isolate RO1-1 and the incompatible *M. oryzae* isolate C9240, as determined by RT-qPCR. ddH_2O was used as a mock-inoculation control, and rice *UBIQUITIN* (*UBQ*) was used as a reference gene to normalize gene expression. Data shown as means ± SD (*n* = 3).

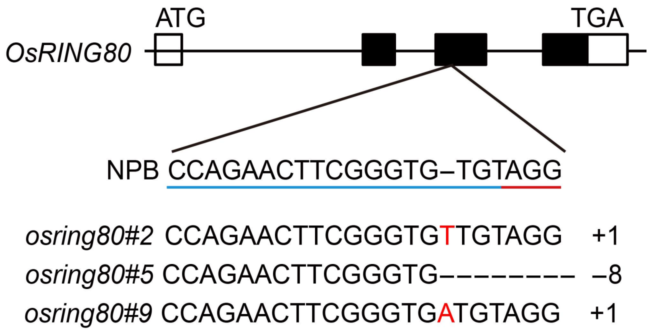

Supplementary Figure S6. CRISPR/Cas9 mediated mutation of *osring80*.
The sgRNA target sequences and PAM motif are underlined in blue and red, respectively.

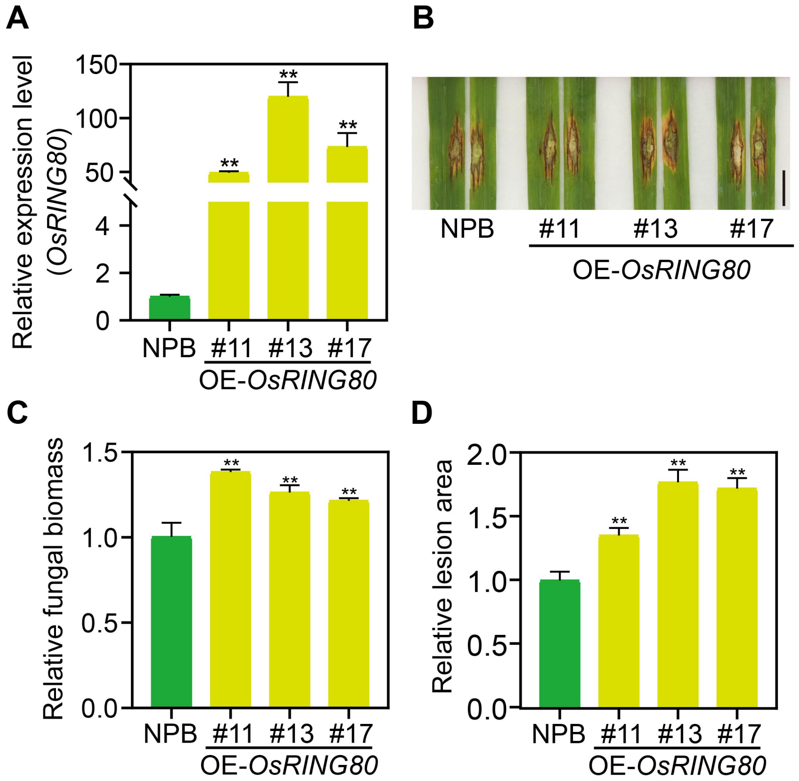

Supplementary Figure S7. *OsRING80* negatively regulates rice immunity against *M. oryzae*.

A) Relative expression level of *OsRING80* in Nipponbare and transgenic plants overexpressing *OsRING80*. B-D) Phenotypes of leaves from 6-week-old OE-*OsRING80* plants inoculated with the compatible *M. oryzae* isolate RB22 (B), relative fungal biomass, as determined by RT-qPCR (C), and the relative lesion area, as measured using ImageJ (D). Data shown as means ± SD (n = 3). Asterisks represent statistically significant differences between the samples (**P < 0.01 by a Student's t-test). Bars: 1 cm.

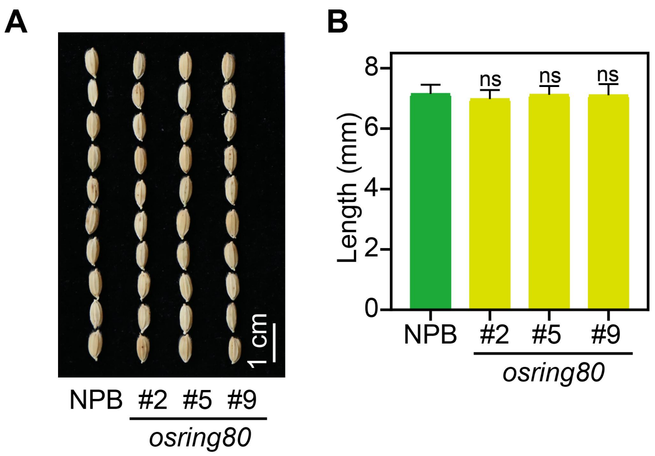

Supplementary Figure S8. The grain length phenotype of the *osring80* mutant.
A-B) Comparison of grain length between Nipponbare plants and the *osring80* mutant. Data shown as means ± SD (n = 20), "ns" indicates no statistical significance at $P > 0.05$ according to Student's t-test.

Supplementary Table S1. Sequences of primers used in this study.

Primers Name	Primers sequence
**Yeast two-hybrid**	
AD-DEP2F	GGAGGCCAGTGAATTCATGGAGCCCGACGCCCCGCTCGA
AD-DEP2R	CGAGCTCGATGGATCCTCACCTGAGCCTTGCATCACCC
AD-DEP2NT(1-690aa)F	GGAGGCCAGTGAATTCATGGAGCCCGACGCCCC
AD-DEP2NT(1-690aa)R	CGAGCTCGATGGATCCAAGGAACAGCATTTCCATCACT
AD-DEP2CT(691-1365aa)F	GGAGGCCAGTGAATTCTGGAGCAGACTAAGAGACCGACAA
AD-DEP2CT(691-1365aa)R	CGAGCTCGATGGATCCCCTGAGCCTTGCATCACCC
BD-OsRING80F	CATATGGCCATGGAGGCCGAATTCATGGCCTCAGTTACTTATATTG
BD-OsRING80R	AGGTCGACGGATCCCCGGGAATTCTCACTGTTCCTTCCAAATTCTCC
**BiFC**	
P2YC-DEP2F	CATTTACGAACGATAGTTAATTAAATGGAGCCCGACGCCCCGCTCGA
P2YC-DEP2R	CACTGCCACCTCCTCCACTAGTTCACCTGAGCCTTGCATCACCC
P2YN-OsRING80F	CATTTACGAACGATAGTTAATTAAATGGCCTCAGTTACTTATATTG
P2YN-OsRING80R	CACTGCCACCTCCTCCACTAGTCTGTTCCTTCCAAATTCTCC
**Pull down**	
MBP-DEP2CF	AAGGATTTCAGAATTCATGGAGCAGACTAAGAGACCGACAA
MBP-DEP2CR	CGACTCTAGAGGATCCTTACCTGAGCCTTGCATCACCC
GST-OsRING80F	GGTTCCGCGTGGATCCATGGCCTCAGTTACTTATATTG
GST-OsRING80R	GTCGACCCGGGAATTCTCACTGTTCCTTCCAAATTCTCC
**Co-IP**	
DEP2-FlagF	AACACGGGGGACTCTAGAATGGAGCCCGACGCCCCGCTCGA
DEP2-FlagR	CTTGTAGTCCATGTCGACTCACCTGAGCCTTGCATCACCCA
OsRING80-GFPF	CGGGTGAGCTCGGTACCAAGCTTATGGCCTCAGTTACTTATATTG
OsRING80-GFPR	AGCGGCCGCACTAGTAAGCTTCTGTTCCTTCCAAATTCTCC
**RT-qPCR**	
q-DEP2F	GACACAAGCTGATCAAGGTGCAT
q-DEP2R	GAACTGCGCTGGGTTCTGGTGG
q-OsRING80F	GAATGTCCAGTCTGCCTGAGCG
q-OsRING80R	CATGGAAGTTCAAGCGACGCAG
q-MoPot2-F	ACGACCCGTCTTTACTTATTTGG
q-MoPot2-R	AAGTAGCGTTGGTTTTGTTGGAT
q-gUBQF	TTCTGGTCCTTCCACTTTCAG
q-gUBQR	ACGATTGATTTAACCAGTCCATGA
q-OsPR1aF	GGAAGTACGGCGAGAACATC
q-OsPR1aR	TGGTCGTACCACTGCTTCTC
q-OsWRKY45F	GCCGACGACCAGCACGATCACC
q-OsWRKY45R	ACGAGCCGACGCCGCCCTC

Supplementary Table S2. Antibodies used in this study.

Antibodies	Source	Identifier
Mouse monoclonal anti-MBP	Abbkine	Cat# A02070-2
Mouse monoclonal anti-GST	BGI Genomics	Cat# AbM59001-2H5-PU
Monoclonal anti-GFP	MBL	Cat# 598-7; RRID:AB_10597267
Monoclonal anti-Flag	MBL	Cat# M185-7; RRID:AB_2687989
Mouse monoclonal anti-Actin	ABclonal	Cat# AC009; RRID:AB_2771701
